# Videoconferencing for Health Care Provision for Older Adults in Care Homes: A Review of the Research Evidence

**DOI:** 10.1155/2017/5785613

**Published:** 2017-09-10

**Authors:** Louise Newbould, Gail Mountain, Mark S. Hawley, Steven Ariss

**Affiliations:** ^1^Centre for Assistive Technology and Connected Healthcare and School of Health and Related Research, The University of Sheffield, Regent Court, 30 Regent Street, Sheffield S1 4DA, UK; ^2^University of Bradford, Bradford, UK

## Abstract

A scoping review was conducted to map the research evidence on the use of videoconferencing for remote health care provision for older adults in care homes. The review aimed to identify the nature and extent of the existing evidence base. Databases used were Embase, Medline, Web of Science, and Cochrane Library Reviews. The review identified 26 articles for inclusion, of which 14 were case studies, making the most used study design. Papers described videoconferencing as being used for assessment, management of health care, clinical support, and diagnosis, with eight of the papers reporting the use of videoconferencing for more than one clinical purpose. A further eight papers reported the use of videoconferencing for assessment alone. The literature reported the collection of various types of data, with 12 papers describing the use of both qualitative and quantitative data. The outcomes mainly addressed staff satisfaction (*n* = 9) and resident satisfaction (*n* = 8). Current evidence supports the feasibility of videoconferencing in care homes. However, research needs to be undertaken to establish the contexts and mechanisms that underpin the successful implementation of videoconferencing in care homes and to define useful measures for success.

## 1. Background

Care homes are defined by the English Care Quality Commission (CQC) as homes that “offer accommodation and personal care for people who may not be able to live independently, with some homes offering 24-hour care from qualified nurses” [1]. According to Gordon et al. (2014) around half of care home residents need help to mobilise, half are incontinent, and three-quarters have dementia [2]. The same research showed that health care provision to care homes in the UK is often inadequate in meeting residents' needs [2]. Earlier work by the Care Quality Commission [3] reported that care home residents often have inadequate access to health care services. It has been suggested that technology may be one way of addressing the problem [4].

A range of digital technologies have already been used for health care purposes in care homes, for example, telemonitoring devices [5–7], telecare devices [8–19], teleconferencing (the use of telephone) [20–23], electronic health care records [24, 25], telepresence devices (remotely controlled robots designed to give a sense of someone being in that location) [26, 27], digital pen and paper technology [28], and teleconferencing and audit feedback [29, 30].

This review focuses on one type of digital technology, videoconferencing. It has been suggested that videoconferencing may be one way of addressing problems with access to health care [4] by improving access to a range of services [31]; encouraging continuity of care [32]; removing the inconvenience of travel [33]; and improving access for those who may have physical disabilities [34]. The purpose of this review was to identify the extent and nature of available research evidence for the use of videoconferencing as a method of health care delivery for older adults in care homes. The review aimed to chart the following characteristics: the clinical purposes for which videoconferencing is being used in care home settings; the countries the research originates from; the research designs used; the types of data collected; and the main outcomes that the research sought to examine.

## 2. Method

The chosen review method was a scoping review [35]. Scoping reviews allow research to be mapped to explore what literature is available, to address a broad research question and establish whether or not a full systematic review would be worthwhile. It focuses on the breadth of research available on a specific topic [36]. This method was selected to identify the extent and nature of evidence currently available [37]. Arksey and O'Malley (2005) identified five key stages to a scoping review; these are as follows: (1) develop the research question; (2) identify studies; (3) select studies; (4) chart the data; and (5) report synthesised results [37]. These stages were followed to address the following research question: what is the extent and nature of available research evidence for videoconferencing as a method of health care delivery for older adults in care homes?

Search terms were identified for the target population and videoconferencing [36] by conducting a preliminary broad search to identify relevant keywords and terms. The terms were also informed by the inclusion/exclusion criteria, which were as follows.

### 2.1. Inclusion

Inclusion criteria were papers that focused on videoconferencing for older adults in care homes, nursing homes, long-term care facilities, and homes for the aged or in residential care.

### 2.2. Exclusion

Exclusion criteria were papers that focused on technical architecture or cost or were aimed at the treatment of people <65; where the article was not available in English; and where the full text could not be acquired. Abstracts were screened to exclude papers where the results were aggregated with those derived from other settings, so the findings for long-term care could not easily be extracted; this included reviews. Where there were duplicate papers of a primary study, only the paper that had the most comprehensive information was included. Opinion pieces were also excluded.

Databases searched were Embase, Medline, Web of Science, and Cochrane Library Reviews. This was followed by a refined search using keywords and terms that were identified through existing literature. When using the database search fields, key words used to define the care home population were limited to the main topic of the article, intervention terms limited to the title, and publication date from 2000 to present day; this was due to the initial search highlighting a lack of relevant research prior to 2000. Search terms used were as follows.

#### 2.2.1. Population

Care home^*∗*^ OR Nursing home^*∗*^ OR Residential care OR Residential facility^*∗*^ OR Long term care OR old-age^*∗*^ home OR old age^*∗*^ home OR residential age^*∗*^ care OR long-term care.

#### 2.2.2. Intervention

Video conference^*∗*^ OR videoconference^*∗*^ OR Video OR videoconsult^*∗*^ OR video consult^*∗*^ OR videoteleconferenc^*∗*^ OR video-teleconferenc^*∗*^

AND

E-health OR telehealth OR telecare OR interactive health communication OR teleconference^*∗*^ OR teleconsultati^*∗*^ OR telemonitor^*∗*^ OR telepresence OR telediagnosi^*∗*^ OR telesurveillance OR technology enabled care services^*∗*^ OR digital health OR telemedicine.

Reference lists of included papers were searched for relevant further papers. Additional published evidence was identified by contacting experts in the field. Key experts at Airedale telehub (a provider of videoconferencing for care homes in North Yorkshire, UK) were contacted along with other sites that were known to have trialled videoconferencing in different contexts.

Of the included papers, 25% were checked by the second author, to validate the selection. The level of agreement was high, with only one paper being excluded as a result of validation. Both authors agreed that the paper did not fit the criteria for inclusion upon discussion.

## 3. Results

**Figure 1:**
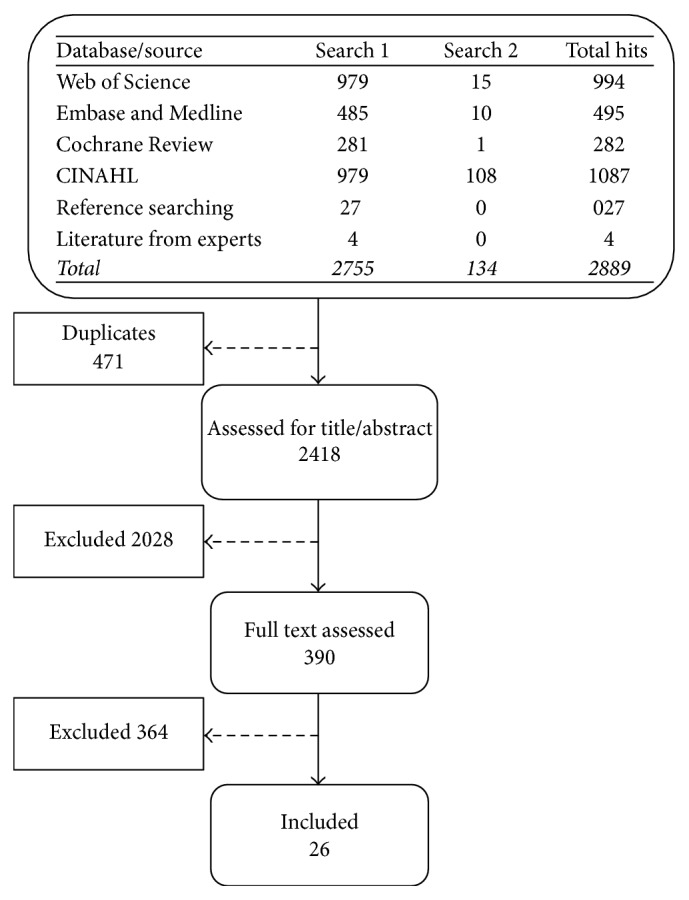
Prisma of search results.

A total of 2889 articles were identified; duplicates were removed (*n* = 471) leaving 2418 to be screened by title and abstract (*n* = 2028 removed) (Figure 1). This left 390 to be screened by full text (*n* = 364 removed), resulting in 26 articles being identified for inclusion in this review.

Data were extracted from the papers based on clinical purpose for using videoconferencing, which countries the research originated from, the study design, type of data collected, and outcomes reported.

### 3.1. Clinical Purpose of Use

Table 1 shows the papers grouped by purpose of use. Eight papers reported the use of videoconferencing solely for health assessment, including wound assessment [38, 39]; assessing clinical changes in dementia patients [40]; general geriatric assessment [41]; assessments by allied health care professionals (dietetics, occupational therapy, physiotherapy, podiatry, and speech pathology) [42]; psychiatric assessments [43, 44]; and assessment of acute medical problems (mental status, abnormal laboratory values, or falls) [45].

Five research papers reported the use of videoconferencing for managing a clinical condition, through a health care professional based at a remote site, such as a hospital [46–50], for example, mental health problems [50].

Two papers did not specify the purpose of use; one used secondary data to establish what health care specialists/doctors had been contacted via the system [33] and the second examined the relationship between the care home and technology provider and how this influenced the outcomes of videoconferencing [51].

In two papers that described videoconferencing being used for clinical support [52, 53], advice was sought by professionals from the remote site, with one paper examining reduction of hospital admissions in residents with Chronic Obstructive Pulmonary Disease (COPD) [52] and the other describing the use of videoconferencing to access a range of health care specialists based at one hospital. Specialists included rehabilitation doctors and orthopaedic surgeons [53]. One paper assessed the use of videoconferencing for diagnosis and its effectiveness in identifying undiagnosed dementia in residents [54].

Eight papers recounted research that had evaluated the use of videoconferencing for more than one purpose [31, 32, 55–60], for example, both assessment and management [60]. Other combinations included assessment and treatment, patient education, management, and falls prevention [31]; diagnosis and developing a treatment plan [32, 56]; assessment and treatment [55]; assessment, review, prescriptions, and follow-up [57]; treatment, prescriptions, advice, referrals, and follow-up [58]; updating a remote team, reviewing care needs, and developing care plans [59]; follow-up and urgent review [60]; and teleeducation, telecounselling, and telemedicine [38].

### 3.2. Country of Origin

Table 2 shows the papers grouped by country of origin. Twelve of the identified papers originated from the USA [32, 38, 39, 43, 45, 48–51, 54, 55, 59]; five were from China [31, 44, 53, 56, 60], three were from the UK [47, 52, 58], and three from Australia [33, 42, 57]. The remaining three papers were from Korea [40], Sweden [46], and France [41].

### 3.3. Study Designs Identified

Table 3 shows the breakdown of papers by study design. The most frequently reported method was case studies, with 14 of the papers describing the use of this design [31, 33, 38, 44, 45, 47–51, 55, 57, 59, 60]. Five cohort studies were identified looking at general practitioner adherence to assessments undertaken during consultations [41], videoconferencing for the diagnosis of dementia [54], and the use of 24-hour consultations [52] and for the care of dementia patients in Korea [40] and one looking at the implementation of videoconferencing in long-term care [53]. There were three studies that used repeated measures, comparing face-to-face contact with videoconferencing [42, 43, 56]. One compared psychiatric assessments [43], another allied health assessments [42], and a third considered podiatric intervention [56]. There was only one randomised controlled trial that examined whether videoconferencing could reduce hospitalisations [39].

### 3.4. Type of Data

Table 4 shows the types of data collected in studies included in this review. The most popular, in 12 studies, was the combination of both qualitative and quantitative data [31, 32, 38, 40, 42, 44, 45, 49, 55–57, 59]: 7 of these included a satisfaction questionnaire and qualitative clinical data [31, 32, 38, 42, 45, 56, 57] such as care records [32] or care plans being reviewed [42]. One paper used clinical outcome scales and observation [49].

Ten of the papers used purely quantitative data [33, 39–41, 47, 50, 52–54, 58]. Secondary data included the use of postcode data to compare admission rates between care homes with and without telemedicine [58] and papers that reported on consultation records and electronic billing [33]. One collected primary data and secondary data to look for trends in areas such as cost reductions [55]. One paper collected routine data and compared nontelemedicine users to telemedicine users to look at admission rates and cost [39] or to compare to other models of long term care [53].

Three of the studies were completely qualitative in nature [46, 48, 51]. For example, one interviewed nursing staff and explored factors that increase the perception of presence [46].

One used only clinical outcome measures [43], aiming to establish whether or not psychiatric assessments could be carried out reliably using videoconferencing [43].

### 3.5. Outcomes

Table 5 shows papers which examined a broad range of outcomes relating to videoconferencing. Most papers considered staff satisfaction when using videoconferencing, with nine papers referring to this in their findings [32, 38, 44, 45, 48, 50, 56, 57, 60]. Eight papers addressed resident satisfaction [32, 38, 44, 45, 48–50, 56, 57, 60], with another eight examining the effect on cost [39, 44, 49, 52, 55, 58–60]. Four of these considered how reduction in admissions reduced cost [39, 52, 58, 59], one addressed how reduction in admissions and in transportation costs to A&E had reduced cost [60], two reported on how reducing visits to outpatient clinics affected cost [44, 55], and one paper considered at how improving the management of Parkinson's through videoconferencing reduced cost spent on medication to manage the symptoms and transportation costs to outpatient clinics [49]. Further seven papers addressed resident outcomes [32, 40, 45, 49, 53, 58, 59]; six examined changes in admission rates [39, 52, 53, 57–59] and feasibility of use [31, 38, 42, 44, 50, 60]. Outcomes that were present in three papers or less were excluded from this table.

## 4. Discussion

The purpose of this review was to identify the extent and nature of research evidence for the use of videoconferencing as a method of health care delivery for older adults in care homes.

This review identified videoconferencing as being most frequently used for clinical assessment, either on its own [38–45], or in combination with other applications [31, 32, 55–60]. There are a wide range of other applications that need to be explored further in future research. For example, this paper highlights a lack of research on the use of videoconferencing for clinical support [52, 53] and diagnosis [54]. Research addressing how the needs of older adults living in care homes affect the range of purposes videoconferencing is used for would also be beneficial in determining how best to apply videoconferencing to meet residents' needs.

The majority of research originated from USA [32, 38, 39, 43, 45, 48–51, 54, 55, 59] and China [31, 44, 53, 56, 60] and three were from Australia [33, 42, 57]. These countries may be more invested in researching videoconferencing, due to the fact that they have large, sparsely populated areas, where remoteness and increased travel time make conventional services more difficult to provide. This may mean that services are more difficult to access and community services may be more challenging to provide, due to the time it would take to travel to remotes services or care homes, in addition to the cost of travelling [47]. Research from other countries was limited. There needs to be more research globally, to gain a better understanding of how videoconferencing would work in different contexts, as the research identified in the review may have limited generalisability to other countries [61].

This review found that very little population-based evidence is available about the use of videoconferencing, with 20 of the papers describing small scale studies, recruiting just one care home [31–33, 38, 42–51, 53–56, 59, 60]. There were only two large studies [52, 58], one of which included 14 care homes [52] and another which included 50 care homes (23 homes without telemedicine, compared to 27 with telemedicine) [58]. In the other studies, recruitment ranged from two to 11 care homes [39–41, 57]. This suggests that research into videoconferencing for remote health care provision in care homes is still in its infancy globally. Additionally, a lack of large controlled studies makes the findings hard to generalise [62].

The most frequent type of data identified in this review was a combination of quantitative and qualitative data (mixed methods) [31, 32, 38, 40, 42, 44, 45, 49, 55–57, 59], suggesting that many studies found it important to look at a range of clinical outcomes as well as exploring stakeholder experiences of using videoconferencing. Although using mixed methods can help address a broader range of research questions and may be unable to capitalise on the strengths of both methods, unless carried out by a large research team, the value of mixed methods approaches requires further investigation. Thus, more purely qualitative or quantitative research may be beneficial to get a more in-depth or broader understanding than may be possible when trying to balance the two approaches [63]. There were only three papers that were completely qualitative in nature [46, 48, 51] meaning that more robust qualitative studies are required, to determine how experiences of using videoconferencing may vary geographically and by purpose of use.

The most frequently reported outcome was staff satisfaction, with fewer looking at resident outcomes or the feasibility of videoconferencing. This suggests that one of the main motivating factors for videoconferencing implementation is to improve staff satisfaction. More robust studies in this area, in addition to further exploring how resident satisfaction and the feasibility of videoconferencing may vary by context, would be beneficial.

The findings from this review highlight a need for more research exploring clinical purposes for videoconferencing in care homes such as for rehabilitation [64]. More research also needs to be conducted globally to get a better understanding of how videoconferencing might work within different clinical and geographical contexts and with different populations of care home residents. Larger controlled trials would help identify the effectiveness of videoconferencing for improving resident's health care. Additionally, more theory driven research is required to identify the mechanisms for change that lead to successful implementation of videoconferencing in care homes. Research designs that have a greater emphasis on rigorously conducted qualitative research would also be useful in terms of getting a more in-depth understanding of the user experience, particularly around resident outcomes and to look more specifically at the reliability and feasibility of videoconferencing in care homes.

## 5. Limitations of the Review

Resources restricted the extent of cross validation of papers for inclusion.

## 6. Conclusions

It is evident from undertaking the scoping review that a systematic review would not be fruitful due to the lack of rigorous studies [37].

The findings show that there are a wide range of applications for videoconferencing technology in care homes, with the most common being for assessment of resident health. Additionally, most of the research was identified as originating from countries that have large, sparsely populated areas.

In order to understand the contexts and mechanisms that lead to successful implementation of videoconferencing in care homes, more vigorous studies need to be undertaken to start to understand outcome patterns that will lead to success or failure of videoconferencing within care homes in different contexts globally.

## Figures and Tables

**Table 1:** Papers grouped by clinical purpose of use.

Clinical purpose of use	Papers
Assessment	8
Management	5
Clinical support	2
Diagnosis	1
Various	8
Not specified	2
*Total*	*26*

**Table 2:** Papers grouped by country of origin.

Country of origin	Papers
America	12
China	5
UK	3
Australia	3
Korea	1
Sweden	1
France	1
*Total*	*26*

**Table 3:** Designs of identified studies.

Main designs	Papers
Case studies	14
Cohort	5
Repeated measures	3
Randomised controlled trials	1
Interviews	1
Observational	1
Cross-sectional	1
*Total*	*26*

**Table 4:** Data reported in papers.

Type of data	Papers
Qualitative and quantitative	12
Quantitative only	10
Qualitative only	3
Clinical outcomes only	1
*Total*	*26*

**Table 5:** Papers grouped by outcomes examined. Papers may appear in more than one category if they discuss more than one of the following.

Outcomes	Papers
Staff satisfaction	9
Resident satisfaction	8
Cost	8
Resident outcomes	7
Admissions	6
Feasibility	6
